# Transposition of Certain Functions of the Teeth

**Published:** 1884-11

**Authors:** J. Edw. Line

**Affiliations:** Rochester.


ARTICLE III.

### ON THE TRANSPOSITION OF CERTAIN FUNCTIONS OF THE TEETH.

BY J. EDW. LINE, D. D. S., M. D. S., ROCHESTER.

A glance at the species, varieties, individuals of the animal kingdom, is sufficient to convince even the casual observer that teeth have several functions, the chiefest of these being what is conceded in the case of most, if not all animals, the mastication of alimentary substances. And by mastication we do not mean merely the trituration, but also the seizure of food, its laceration, division, which are but a few of its many modifications. Animals whose food is derived from the vegetable kingdom grind, triturate, reduce to a fine, and in some cases the finest possible, condition all food fit for alimentation and whose appointed end is the maintenance of their own economy. It is retained, as a rule, in the cavity of the mouth until its every particle has been exposed to the mechanical action of the teeth and the solvent effects of the secretion of the salivary glands. And this, in the case of the vegetable feeders, is a very necessary process ; for without thorough mastication and insalivation, the reduction of the starchy matter in which such food abounds would be incomplete, and as consequences of such incomplete reduction, other and remote organs would be required to perform work the quantity and quality of which would overtax them and their resources; or the food would be rejected or voided nearly or quite, as replete in nutritious ingredients as when first ingested. In the case of the flesh-eaters the insalivation of food is of little consequence further than the wetting, oiling, greasing of the mass to aid it in its passage to the more distant digestive organs; but the tearing, cutting, in a word full of meaning, the *mangling* of the food, is of the greatest moment; for by this process the surface subsequently exposed to the secretions of the several organs is many times multiplied and the mass more readily acted upon preparatory to its assimilation. It is thus seen that mastication is an important function, and the further pursuit of the matter would add little to the plain statement, that so far at least as the lower animals are concerned, work is the primary function of teeth and those other parts that go to make up the dental apparatus. But teeth have other functions which are said by the comparative anatomist to arise "out of the relations of co-existence with other organs and endowments, or from a special development of the organs themselves." These functions are termed secondary, and may be briefly mentioned as exhibiting themselves in speech, as ornaments, as discoverers of age, as characterizing sex, as weapons of offence and defence, as a means of anchorage, as implements of tranport (—*Owen*); but the greatest of these, greater even than mastication, in the civilized member of a civilized community is ornament or personal adornment.

But before going further, and that we may get at the subject in form for discussion, we submit the following propositions: 1st.—Among animals, other than man, mastication, including the several modifications already alluded to, is the primary function of teeth. 2d.—Among a very small proportion of animals other than man, and in fact including the lower orders of men, ornament or personal ornament is a secondary function.

Enough has been said upon these two points to show much, if not everything, in their favor, hence their dismissal here and now. Then we have 3d.—In civilized communities of men ornament or personal adornment is the primary, and mastication a secondary function. 4th.—This transposition of function is more or less complete, and always directly as the civilization of the individual and the com munity of which he is a factor.

That our third proposition is true we cite a number of facts that as dentists we are daily compelled to face: In the first place we meet with children to the care of whose teeth not a thought is given by the parents till the anterior teeth show the familiar green stain, begin to decay, or melt away before solvent secretions the nature of the action which is much guessed at, but continues as yet quite unknown. The thought of looking into the child's mouth to note the condition of teeth ordinarily out of sight, unless lead to it by an aching member, occurs to so few parents that each of us could, in all probability, count such families in our practice once around the fingers of both hands. That it is looks, not use, that is uppermost in the parents' mind, is evident from the frequency of the request to remove the stain from the child's front teeth,—"just those four, you know." How the child eats is of secondary importance; how he looks, primary and vital.

Again, we are called upon by, say a well-dressed and (her teeth excepted) refined (?) woman, with the request to examine her teeth preparatory to an appointment. Her teeth are clean, as clean as a stiff brush and coarse (or fine, as the case may be) powder vigorously applied at long intervals, can make them; but with this clean state of the teeth we find abnormally red and swollen gums, with patches of lacerated tissue dipping down between the teeth. At the first view of the case we say—Hard brushing at long intervals, rather than the better rule of little and often. Her occasional respect for herself, and the commendable desire to show us a little of that same commodity, are appreciated at their full value; but the words all but escape us, "Brushed for the occasion." At home, no thought of teeth if they give no trouble ; abroad, they must correspond with articles especially donned for show ; their function for the time being is ornament or personal adornment. And again, how often do we hear the half-formed determination to let fillable or irregular, but otherwise good teeth go, that their owners may confessedly obtain artificial teeth free from the blemishes of decay, or misplacement in the alveolar portions of the jaw. And who among us has not from time to time extracted by request a somewhat troublesome but fillable back tooth, simply because it was a back tooth, and for a person who would suffer for days, perhaps weeks, rather than part with one from the anterior portion of the jaw ? The almost daily request to fill the front teeth with gold and the back ones with something cheaper, is off the same piece—teeth primarily for looks, incidentally for use.

Our fourth proposition, that this transposition of function is directly as the civilization of the individual, seems to us to be closely related to the self-evident. Among members of the lower orders of men teeth are as, a rule, regarded as of value because of their usefulness in preparing for further digestion and for assimilation the various articles used as food ; and the more labor involved in the preparation of such articles of food, the greater the value attached to these with us much abused organs. To repeat—low down in the scale of civilized beings teeth are valued for what they do, and not for what they appear ; but as we ascend in the scale, less and less importance is attached to their usefulness, and more and more to their investment with what belongs to decorative art. It will thus be seen—must be seen, it seems to us—without any attempt at argument, that teeth when viewed from the utilitarian standpoint, belong to the savage and his compeers in civilized life who have not yet arrived at angelhood ; while from the æsthetic standpoint their primary function ranks with human hair. In other words, and to repeat our proposition—this transpositoin is directly as the civilization of the individual and the community in which he lives, and is becoming more marked day by day.

Having stated the case, it is now no more than fair for us to indicate the causes that have contributed, and are still contributing, to this change. It is not necessary to go back to the physical and mental activities that have led to the invention of reducing and refining apparatus and methods, whereby the food of civilized man is prepared for the stomach long before it reaches his mouth. We have this product at hand, and *must* use it—must, for there is no alternative. We would not go back to the ways of our remote ancestors, nor even to those of some of our unfortunate neighbors; and more than that, the demands of modern society are such that we could not do so if we would. Vegetable food is softened and chemically changed by cooking, so that to be saturated with saliva for digestive purposes, it needs only to be retained in the mouth a short time, rolled from side to side by lips, cheeks and tongue, and occasionally mashed by the ever-movlng lower jaw; while animal food, in addition to its being thoroughly cooked, is cut, sliced, shaved, chopped, by the help of a knife and fork, till the surface made ready for the direct and solvent action of the gastric juice, is commensurate with that of a plate of that mysterious something very properly called hash. Here we have not a division of labor, but worse—worse for the teeth,—a shouldering of all labor, first on the furniture and methods of the kitchen, and secondly, on the stomach and more remote digestive organs—the mouth and its dental contents being a mere hole in the wall, a sort of rapid transit from Bridget to belly. In some cases there is compensation for this—for instance, in those persons who grind their teeth at night, or while at work during the day; and also in the educated tobacco chewer, the preservation of whose teeth is due less to the antiseptic effects of the weed than to the exercise necessary to its artistic manipulation. It is thus seen that work is essential to the making of good teeth, also to the keeping of them good. Exercise makes a tooth, as it does any other organ, hungry. Hunger means a right condition of the tooth to appropriate a right quantity of a quality of nutritive material, and that a tooth in this state feed itself, it is only necessary to place this material within the tooth's reach. This view of the matter may be fairly regarded as an illustration of the principle of natural selection—the animal as conditioned by his environment, teeth in their relations to the work they are called upon to do and the nutritive material by which they are fed and sustained.

But there is another cause, or set of causes, that plays a part in this transposition of function. and which falls under the head of sexual selection. The male element in the community, and quite as often or oftener, the female, marries for wealth, position, influence; but never without taking into consideration the personal make-up of the other person. Blemishes of physique, or of mind, are always against the person; but not necessarily to the extent of his or her rejection. Except for the advantages named, a representative of either sex would not, other things being equal, pair with an actually, or even prospectively, toothless representative of the other sex, if the choice lay between teeth and toothlessness. A young girl without incisor teeth of some kind, is, from an æsthetic standpoint, unsightly, unattractive, and as such would be severely let alone by the marriageable and marrying young buck, provided he could find a toothsome and toothful young girl who could, in this and in all other respects, meet the requirements of his special case. The result in this instance would be a strain from ancestors possessing teeth, with reasonable hope of teeth in the offspring; while in the case of said young buck and the toothless girl, the progeny would inherit from the parents a tendency to teeth modified by the further tendency to early loss. But our young girl without teeth, or with bad teeth, is equal to the emergency—is not to be left; and knowing some of the advantages of a good looking mouth, and how much sometimes depends upon its possession, she applies to us for that she has not, or for better than she has, carries out the deception, propagates her kind, and hastens the day of the extinction of those parts of the human frame, repairs on which enable us to provide for ourselves and those dependent upon us the ever welcome bread and butter.

A recapitulation of what we have said leads to conclusions somewhat as follows: that mastication was, and with some grades of men is, the primary function of teeth; that ornament or personal adornment was a secondary function of human teeth; that in civilized communities this order is now reversed—ornament or personal adornment having become primary, mastication secondary; that this transposition of function is directly as the civilization; that the causes leading to the suppression of mastication are such as fall under the head of natural selection; that the causes leading to the advancement of a secondary function to the first place in the list of functions of the teeth are attributable, in the main to several selections, incidentally to the talent and talent and genius of the dental profession.—*Odontographia Journal.*

